# Supplementary material for: Ultrathin Washcoat and Very Low Loading Monolithic Catalyst with Outstanding Activity and Stability in Dry Reforming of Methane
Source: Nanomaterials (Basel). 2020 Mar 1;10(3):445. doi: 10.3390/nano10030445 (PMC7152993; doi:10.3390/nano10030445)
Supplement: Supplementary file 1 [file nanomaterials-10-00445-s001.pdf]

Supplementary Materials

Ultrathin washcoat and very low loading monolithic catalyst with outstanding activity and stability in dry reforming of methane

Fazia Agueniou, Hilario Vidal, M. Pilar Yeste, Juan C. Hernández-Garrido, Miguel A. Cauqui, José M. Rodríguez-Izquierdo, José J. Calvino and José M. Gatica

Departamento de Ciencia de los Materiales e Ingeniería Metalúrgica y Química Inorgánica. Facultad de Ciencias, Universidad de Cádiz, Campus Rio San Pedro, Puerto Real, Cádiz-11510, Spain

IMEYMAT, Instituto Universitario de Investigación en Microscopía Electrónica y Materiales, Universidad de Cádiz, 11510 Puerto Real, Spain.

Synthesis of the powdered and monolithic catalysts

The ceria-zirconia support was prepared from a commercial nano-sized zirconia from Tecnan-Nanomat S.L (78 $m^2 g^{-1}$ and 10-15 nm of BET and average particle size, respectively) which was impregnated to incipient wetness with a $Ce(NO_3)_3{\cdot}6H_2O$ (99.99%, Sigma Aldrich) aqueous solution (0.88 M) to obtain an oxide having a 20%Ce-80%Zr nominal molar composition. After drying in air (110 ^{0}C, 12 h) and grinding, the sample was calcined (500 ^{0}C, 1 h). The resulting oxide was further activated for its redox properties enhancement following a thermo-chemical aging protocol known as SRMO (Severe Reduction-Mild Oxidation) proposed by our lab (Yeste et al., 2013). Briefly, it consists in a reduction treatment under H_2(5%)/Ar (950 ºC, 2 h), followed by evacuation (950 ^{0}C, 1 h) and oxidation with pulses of O_2(5%)/He at room temperature, and finally heating under the oxidising mixture up to 500 ^{0}C (1 h).

The supported nickel catalyst ($Ni/CeO_2/ZrO_2$) was prepared by incipient wetness impregnation with a Ni $(NO_3)_2{\cdot}6H_2O$ (99.99%, Sigma Aldrich) aqueous solution (1.01 M) aiming to reach a nominal 5 wt% metal loading. The catalytic precursor was dried (110 ^{0}C, 12 h), calcined (400 ^{0}C, 1 h) and finally grinded.

Cordierite blocks (Corning) with a cell density of 36 cells cm^{-2} (230 cpsi) and a wall thickness of 0.18 mm (7 mil) were cut to obtain cylindrical pieces having 13 mm of diameter, 47 mm of length and 2 g of approximate weight.

Monolithic catalysts were prepared by washcoating, from a slurry (stabilized at pH 4.0 using acetic acid) containing the $Ni/CeO_2/ZrO_2$ catalyst. The granulometry (d_{90} = 0.80 μm) and the Z potential (36 mV at pH = 4, with the isoelectric point at pH = 6.8) data for this sample are proper for the washcoating method as previously reported (Gómez et al., 2014). Therefore, we prepared slurries containing the nickel catalyst (19.1 wt%), polyvinyl alcohol (1.7 wt%), Nyacol AL20 colloidal alumina (4.2 wt%) and water. As far as our objective was to reach low catalyst loadings, we decided to prepare slurries with low viscosity (5.1 mPa s).

Cordierite pieces were immersed (3 cm min^{-1}) in this slurry, kept fully immersed for 90 s, the first 15 s under ultra-sonication. They were further pulled out at the same rate, and the excess of slurry was removed by air flowing. The pieces were then dried at 120 ^{0}C for 30 min and submitted to one or two new coating/drying cycles until reaching the desired final specific loading, around 0.4 mg cm^{-2} corresponding to a washcoat loading about 25 mg per g^{-1} of support. Finally, all the monoliths were calcined (5 ^{0}C min^{-1}) at 450 ^{0}C (1 h). The reached average active phase loading, estimated from the weight gain after calcination, resulted to be 0.36 mg cm^{-2}. The residual slurry, containing the $Ni/CeO_2/ZrO_2$ catalyst, was dried and submitted to calcination at 450 ^{0}C (1 h) to obtain the reference powdered sample for the catalytic study. This sample was characterized by ICP-AES using a Thermo

Elemental Iris Intrepid equipment obtaining the following results: 3.9 %wt. of Ni content and a 0.18 Ce/Zr molar ratio.

Gómez, D.M.; Gatica, J.M.; Hernández-Garrido, J.C.; Cifredo, G.A.; Montes, M.; Sanz, O.; Rebled, J.M.; Vidal, H. A novel CoOx/La-modified-CeO_2 formulation for powdered and washcoated onto cordierite honeycomb catalysts with application in VOCs oxidation. *Appl. Catal. B* **2014**, *144*, 425–434.

Yeste, M.P.; Hernández-Garrido, J.C.; Arias, D.C.; Blanco, G.; Rodríguez-Izquierdo, J.M.; Pintado, J.M.; Bernal, S.; Pérez-Omil, J.A.; Calvino, J.J.. Rational design of nanostructured, noble metal free, ceria–zirconia catalysts with outstanding low temperature oxygen storage capacity. *J. Mater. Chem. A* **2013**, *1*, 4836-4844.

Experimental details of the catalytic test and thermodynamic estimate

Catalytic performance in the Dry Reforming of Methane (DRM) reaction was evaluated for both powdered and honeycomb monolithic samples. These tests were run in quartz reactors, at atmospheric pressure and using a 1:1 mixture of pure CH_4 and CO_2 as feedstock. For powders, 26 mg of sample were diluted in 52 mg of SiC, adjusting the total flow to 50 mL min^{-1}. In the case of monoliths, we employed 21 mm long pieces, containing 13 mg of catalyst, and a total flow of 25, 50 or 75 mL min^{-1} depending on the Weight Hourly Spatial Velocity (WHSV) selected. Small pieces of quartz at the inlet were included to guarantee a turbulent flow. The reaction temperature ranged from 750 up to 900 ^{0}C. The WHSV, expressed as the ratio between reactants flow and sample amount, was 115, 231 or 346 L g^{-1} h^{-1}, while Time on Stream (TOS) in these experiments varied from 24 to 48 h. In all cases the catalysts were subjected to a reduction pre-treatment with 60 mL min^{-1} of 5%H_2/Ar at 600 ^{0}C (2 h). The reaction temperature was measured by means of a thermocouple located in contact with the quartz reactor at the position of the catalytic bed. The gas analysis at the inlet and outlet of the reactor was performed by gas chromatography (Bruker 450-GC), using helium (25 mL min^{-1}) as carrier inert gas. Reactants (CH_4 and CO_2) conversion values were calculated from the inlet and outlet molar fractions of the individual gases as follows:

$$CH_4\ Conversion\ (\%) = 100 \times \frac{[CH_4]_{inlet} - [CH_4]_{outlet}}{[CH_4]_{inlet}}$$

$$CO_2\ Conversion\ (\%) = 100 \times \frac{[CO_2]_{inlet} - [CO_2]_{outlet}}{[CO_2]_{inlet}}$$

The molar fractions at the outlet of the reactor were previously corrected to account for the volumetric change in the DRM reaction.

The estimated carbon balances were always in the range 102-106%, therefore within the error of determination of the components.

The estimate of thermodynamic conversion limit values was performed using the DETCHEM software (Deutschmann et al., 2018). This values resulting to be the following:

Table S1. CO_2 and CH_4 equilibrium conversion data for the DRM reaction conducted at 1 atm and with CH_4:CO_2 = 1:1 based on thermodynamic analysis

Temperature (^{0}C)	CH_4 Eq. Conv. (%)	CO_2 Eq. Conv. (%)
750	91	94
800	95	97
900	98	99

Deutschmann, O.; Tischer, S.; Kleditzsch, S.; Janardhanan, V.; Correa, C.; Chatterjee, D.; Mladenov, N.; Minh, H.D.; Karadeniz, H.; Hettel, M.; Menon, V. DETCHEM Software package, 2.7 ed., www.detchem.com, Karlsruhe 2018.

Supplementary electron microscopy characterization

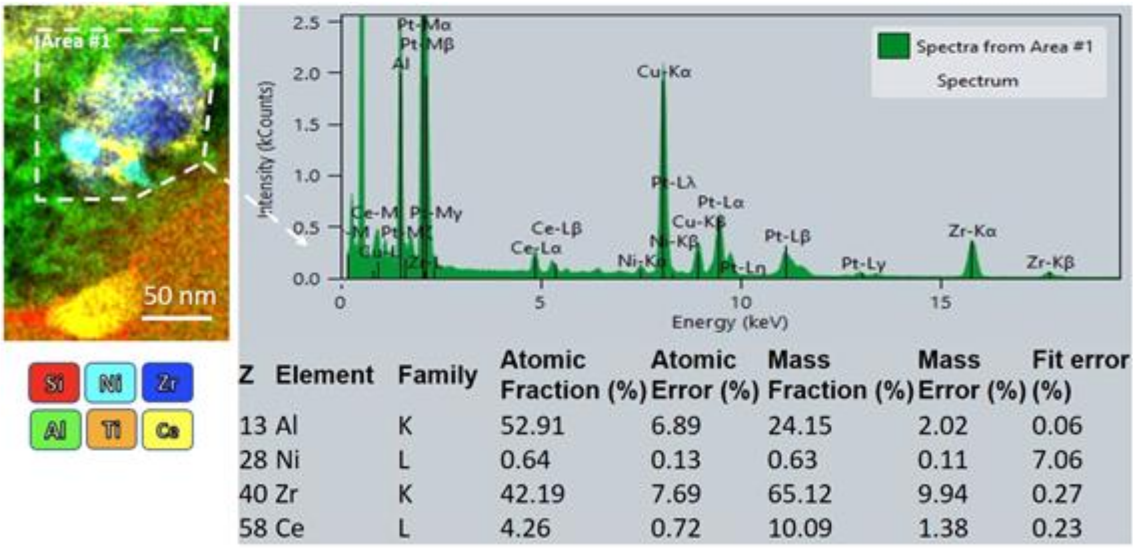

Z	Element	Family	Atomic Fraction (%)	Atomic Error (%)	Mass Fraction (%)	Mass Error (%)	Fit error (%)
13	Al	K	52.91	6.89	24.15	2.02	0.06
28	Ni	L	0.64	0.13	0.63	0.11	7.06
40	Zr	K	42.19	7.69	65.12	9.94	0.27
58	Ce	L	4.26	0.72	10.09	1.38	0.23

Figure S1. Quantitative compositional analysis results from a selected area (Area#1) of the catalyst particle shown in Figure 2 including Ni-Ce-Zr elements (Left) and their corresponding EDS spectra. Elemental contents should be taken with caution considering that they correspond to a tiny portion of the washcoat.
